# Deconstructing the Bat Skin Microbiome: Influences of the Host and the Environment

**DOI:** 10.3389/fmicb.2016.01753

**Published:** 2016-11-17

**Authors:** Christine V. Avena, Laura Wegener Parfrey, Jonathan W. Leff, Holly M. Archer, Winifred F. Frick, Kate E. Langwig, A. Marm Kilpatrick, Karen E. Powers, Jeffrey T. Foster, Valerie J. McKenzie

**Affiliations:** ^1^Department of Ecology and Evolutionary Biology, University of Colorado BoulderBoulder, CO, USA; ^2^Departments of Botany and Zoology, University of British ColumbiaVancouver, BC, Canada; ^3^Cooperative Institute for Research in Environmental Sciences, University of Colorado BoulderBoulder, CO, USA; ^4^Department of Ecology and Evolutionary Biology, University of California, Santa CruzSanta Cruz, CA, USA; ^5^Bat Conservation InternationalAustin, TX, USA; ^6^Biology Department, Radford UniversityRadford, VA, USA; ^7^Department of Molecular, Cellular, and Biomedical Sciences, University of New HampshireDurham, NH, USA

**Keywords:** bat ecology, host-associated bacteria, microbiome, molecular ecology, microbial ecology, white-nose syndrome, 16S rRNA

## Abstract

Bats are geographically widespread and play an important role in many ecosystems, but relatively little is known about the ecology of their associated microbial communities and the role microbial taxa play in bat health, development, and evolution. Moreover, few vertebrate animal skin microbiomes have been comprehensively assessed, and thus characterizing the bat skin microbiome will yield valuable insight into the variability of vertebrate skin microbiomes as a whole. The recent emergence of the skin fungal disease white-nose syndrome highlights the potentially important role bat skin microbial communities could play in bat health. Understanding the determinant of bat skin microbial communities could provide insight into important factors allowing individuals to persist with disease. We collected skin swabs from a total of 11 bat species from the eastern United States (*n* = 45) and Colorado (*n* = 119), as well as environmental samples (*n* = 38) from a subset of sites, and used 16S rRNA marker gene sequencing to observe bacterial communities. In addition, we conducted a literature survey to compare the skin microbiome across vertebrate groups, including the bats presented in this study. Host species, region, and site were all significant predictors of the variability across bat skin bacterial communities. Many bacterial taxa were found both on bats and in the environment. However, some bacterial taxa had consistently greater relative abundances on bat skin relative to their environments. Bats shared many of their abundant taxa with other vertebrates, but also hosted unique bacterial lineages such as the class Thermoleophilia (Actinobacteria). A strong effect of site on the bat skin microbiome indicates that the environment very strongly influences what bacteria are present on bat skin. Bat skin microbiomes are largely composed of site-specific microbiota, but there do appear to be important host-specific taxa. How this translates to differences in host-microbial interactions and bat health remains an important knowledge gap, but this work suggests that habitat variability is very important. We identify some bacterial groups that are more consistent on bats despite site differences, and these may be important ones to study in terms of their function as potential core microbiome members.

## Introduction

Host-associated microbial communities and how they interact as symbionts on their hosts remains an incipient area of research (Ley et al., 2008; McFall-Ngai et al., 2013). Many complex factors can influence and drive the host-microbiome relationship, including abiotic factors, biogeography of the host or microbe, host evolutionary relationships, host health, and the presence of other organisms in the system (Martiny et al., 2006; Costello et al., 2009; Grice et al., 2009; Kelly et al., 2014). Much of the research has focused on the gut microbiome, particularly in humans and other animals, given the large role the gut microbes play in digestion, immunity, and health (Ley et al., 2008; Costello et al., 2009; McFall-Ngai et al., 2013). However, the importance of the skin microbiome and its interactions with host tissue and range of functions are just beginning to be recognized (Grice and Segre, 2011; Chen and Tsao, 2013). The skin acts as a barrier between the host and the environment, and is comprised of a highly diverse community of microorganisms that can vary based on time, body location, and disease status, and the habitat of the host (Costello et al., 2009; Grice et al., 2009; Fierer et al., 2010; Song et al., 2013). In wild animals, we are just beginning to understand the composition of the skin microbial community, and studies thus far have included marine fishes (Larsen et al., 2013), amphibians (Fitzpatrick and Allison, 2014; Kueneman et al., 2014), vultures (Roggenbuck et al., 2014), and whales (Apprill et al., 2011, 2014). These studies are important given that the skin microbiome sits at the interface of hosts and their environment, and thus is the first line of protection against many pathogens and provides an opportunity to acquire or disperse microbial symbionts.

The aim of this study is to examine the factors that influence variability in the bat skin microbiome. Bats are the only mammal capable of winged flight and they live in a diverse range of habitats including caves, mines, buildings, trees, and rock crevices, exposing them to a breadth of environments and microbes (Fenton, 1997; Kunz and Lumsden, 2003; Kunz et al., 2011). Across the diversity of bats, social structures vary across species; some bats are solitary, others are colonial, and some cohabit in multi-species complexes (McCracken and Wilkinson, 2000; Kerth, 2008). In addition, bats can have both intra- and inter-species interactions that may permit an exchange of microbial symbionts between individuals. Due to these close interactions across species and possible exchanges between the host and the environment, understanding the bat microbiome is an important, and currently unexplored, area of bat ecology. Temperate hibernating bats in North America are currently threatened by the emerging fungal skin pathogen, *Pseudogymnoascus destructans* (Blehert et al., 2009; Frick et al., 2010; Langwig et al., 2012) which causes white-nose syndrome (WNS) (Lorch et al., 2010; Warnecke et al., 2012) and continues to spread rapidly across North America (USFWS 2016). Previous studies have identified the presence of bacteria with anti-fungal properties against *P. destructans* on bat skin (Hoyt et al., 2015), and bat skin also shows a marked inflammatory response as a result of *P. destructans* infection (Field et al., 2015). Therefore, it is timely to gain an understanding of the drivers that shape the natural bat skin microbiome as it may be a key determinant of colonization and pathogenicity of *P. destructans*.

Bats also provide a new perspective for skin microbiome research due to the many unique characteristics of their integument. Bat skin provides multiple functions to its host, including acting as a means of producing flight, forming the pinnae of large and sensitive ears, and creating unique facial features across multiple species (Quay, 1970). It can be as thin as 30 micrometers in the wing membranes, and two to three times thicker across other areas of the body. This thin exposed skin may also be a source of evaporative water loss (Chew and White, 1960; Herreid and Schmidt-Nielsen, 1966), which can stress hibernating or diseased bats (Cryan et al., 2010; Warnecke et al., 2013; Verant et al., 2014). In addition, the skin must withstand rapid cooling and warming during hibernation when the animals undergo repeated bouts of torpor and euthermia (Geiser, 2004, 2013). There are also many types of glands within the integument (i.e., sebaceous, sudoriferous) that contribute to the maintenance of the skin environment, as well as specialized glandular organs in some species (Quay, 1970). Understanding the host factors influence the microbial community of bats may begin a foundation for understanding how the microbiota affect the host, and its role in host health. Bats also provide a new perspective for skin microbiome research due to the many unique characteristics of their integument. Bat skin provides multiple functions to its host, including acting as a means of producing flight, forming the pinnae of large and sensitive ears, and creating unique facial features across multiple species (Quay, 1970). It can be as thin as 30 micrometers in the wing membranes, and two to three times thicker across other areas of the body. This thin exposed skin may also be a source of evaporative water loss (Chew and White, 1960; Herreid and Schmidt-Nielsen, 1966), which can stress hibernating or diseased bats (Cryan et al., 2010; Verant et al., 2014; Warnecke et al., 2013). In addition, the skin must withstand rapid cooling and warming during hibernation when the animals undergo repeated bouts of torpor and euthermia (Geiser, 2013). There are also many types of glands within the integument (i.e., sebaceous, sudoriferous) that contribute to the maintenance of the skin environment, as well as specialized glandular organs in some species (Quay, 1970). Understanding the host factors influence the microbial community of bats may begin a foundation for understanding how the microbiota affect the host, and its role in host health.

In this study, we will address the following question: how does the skin bacterial community vary across bat species, across different sites and regions, and across infection states (e.g., *P. destructans* positive or negative) in North American bats? We hypothesized that species and environmental differences among sites would be two possible drivers for the bat host-associated microbial community. This study provides an important baseline for understanding what bacterial taxa are observed across a variety of habitats and species, and the ecological drivers of the bat skin microbiome.

## Materials and methods

### Bat sampling

We surveyed five sites in Virginia and New York during the fall of 2011 to collect samples from bats and cave substrates at the start of the hibernation period (Table 1). Sites in both locations in the eastern U.S. were within the white-nose syndrome (WNS) epidemic area at the time of sampling, however no bats showed active infection at the time of sampling and all were tested for the presence of *P. destructans*. In addition, during the summer and fall of 2012 and 2013, bats and cave substrates were sampled from 14 sites across the state of Colorado. Collecting permits were granted for each state (CO, NY, VA) and work was conducted with an approved IACUC protocol (#1305.06 from the University of Colorado). Sampling was conducted opportunistically at different sites (including caves, mines, and buildings) where bat populations or cave habitats were being monitored by different state and federal agencies. We sampled four species of hibernating bats in the eastern United States: the little brown bat (*Myotis lucifugus*), tricolored bat (*Perimyotis subflavus*), northern long-eared bat (*Myotis septentrionalis*), and the Indiana bat (*Myotis sodalis*). In Colorado, we sampled eight species across a variety of habitat types: long-legged myotis (*Myotis volans*), little brown bat (*Myotis lucifugus*), western small-footed bat (*Myotis ciliolabrum*), Yuma myotis (*Myotis yumanensis*), big brown bat (*Eptesicus fuscus*), western long-eared bat (*Myotis evotis*), hoary bat (*Lasiurus cinereus***)**, and Townsend's big-eared bat (*Corynorhinus townsendii*). One species, *M. lucifugus*, commonly inhabited sites in all three regions (CO, VA, and NY), allowing us to assess geographical and environmental effects in greater depth.

**Table 1:** **Summary of samples for experiment**.

**State**	**Sample site**	**Environmental samples**	***M. lucifugus***	***P. subflavus***	***M. sodalis***	***M. septentrionalis***	***C. townsendii***	***M. volans***	***M. ciliolabrum***	***M. yumanensis***	***M. evotis***	***E. fuscus***	***L. cinereus***
Virginia	VA Site #1	–		5		2							
	VA Site #2	2	3										
New York	NY Site #1	4	8		1								
	NY Site #2	4	15		11								
Colorado	Boulder	10											
	Carbondale Cabin	5	9										
	CB Mine	3											
	Canon City	–					2						
	Dotsero	4	1					20					
	DeBeque	–						1	1		1		
	K Mine	1											
	Pueblo	–								23		1	
	Larimar	–	8									18	
	Lake Cabin Carbondale	–	25										
	Littleton	–									1		
	Colorado Springs	5											
	Salida	–	1					3	2				2
Total	17	38	70	5	12	2	2	24	3	23	2	19	2

A summary of the samples collected for the study, across three states, seventeen sites, and eleven species. Lightly shaded cells represent samples used for the paired environment-bat skin subset.

To target the bacterial communities of bat skin as well as the surrounding environment, a sterile swab protocol was established and used across sampling teams. Each bat was individually sampled using a sterile rayon or cotton swab that was moistened with autoclaved (sterile, DNA free) water, and care was taken to use sterile technique to prevent cross-contamination. Bats were captured using either harp or mist nets that were sterilized prior to use at each site. Captured animals were removed from traps and handled with clean, sterile gloves that were changed after every individual. The skin of the bat along the forearm and muzzle were swabbed firmly with the same swab tip five times along each targeted area, for a total of ten passes (Langwig et al., 2015). After skin swabs were taken, the weight, sex, species, and hibernaculum site were recorded for each individual. If access to the roost site was available, environmental samples of roost walls (rock, mine shaft, house) were taken by passing the swab ten times over the surface in areas where bats were roosting. If soil was available in the roost site, small samples were taken directly beneath roosts using a sterilized scoopula and placed in small, sterilized plastic bags and were sampled using swabs for final analysis. In downstream analyses, environmental sample refer to those sites where it was possible to collect any of the above types of samples. Samples were stored on ice until they could be shipped or transported to the University of Colorado Boulder, where they were kept in a −20°C freezer prior to DNA analysis and sequencing.

### DNA extraction and sample processing

DNA extraction, library preparation, and sequencing of the 16S rRNA marker gene was conducted as in Fierer et al. (2012). Briefly, DNA was extracted using the MoBio PowerSoil extraction kit following the manufacturer's protocol. PCR amplification was conducted using the primers 515F/806R to target the V4 region of 16S rRNA and contained 12 bp barcodes as well as Illumina sequencing adapters following Caporaso et al. (2011). The PCR reactions contained 11 μL PCR water, 10 μL 5 Prime Master Mix, 1.0 μL each of the forward and reverse primers, 1.0 μL MgCl_2_, and 1.0 μL genomic DNA. The thermocycling conditions for PCR consisted of an initial denaturation step of 94°C for 3 min, followed by 35 cycles at 94°C for 45 s, 50°C for 60 s, 72°C for 90 s; and final extension of 10 min at 72°C. Each sample was amplified in triplicate and combined. Amplicons were quantified using the Quant-IT Picogreen dsDNA reagent in 1X TE buffer. A composite sample for sequencing was created by combining equimolar ratios of amplicons from the individual samples and was cleaned using the MoBio UltraClean PCR clean up DNA purification kit. The final sample including aliquots of the sequencing primers were sequenced at the Biofrontiers Next-Gen Sequencing Facility (University of Colorado Boulder). Samples collected in the Eastern US (*n* = 55) were sequenced using the Illumina HiSeq platform in 2011. Samples collected in Colorado (*n* = 147) were sequenced using the Illumina MiSeq platform in 2013.

### *Pseudogymnoascus destructans* assay

We tested for *P. destructans* DNA using real-time quantitative PCR following the protocol of Muller et al. (2013). We quantified *P. destructans* based on the cycle threshold (*C*_t_) value to estimate the fungal load on each bat, with a *C*_t_ cut-off of 40 cycles. The standard curve for quantification was generated using genomic DNA from *P. destructans* ATCC MYA-4855 quantified with the Quant-IT PicoGreen double-stranded DNA assay kit (Life Technologies, Carlsbad, CA) in conjunction with a DynaQuant 300 fluorometer (Harvard Bioscience, Inc., Holliston, MA). Serial dilutions of the DNA from 10 ng to 1000 fg were prepared and analyzed with IGS qPCR, resulting in a significant curve from 17.33 to 30.74 *C*_t_ (C_t_ = −3.348^*^(Log10 P. destructans [ng]) + 22.049).

### Data analyses

Both sets of HiSeq (from the eastern US) and MiSeq (from Colorado) sequences were de-multiplexed using a custom python script “prep_fastq_for_uparse.py,” available at: https://github.com/leffj/helper-code-for-uparse). We trimmed the MiSeq forward reads to 100 bp in length (Prober et al., 2015) in order to combine them with the HiSeq data. Both HiSeq and MiSeq sequences were then combined into a single data file. The combined dataset was processed using the UPARSE pipeline (Edgar, 2013). After de-multiplexing, sequences were filtered against a maximum per-sequence expected error frequency value of 0.5 and singleton sequences were removed to filter out low quality sequences. The resulting sequences were clustered at the ≥97% similarity threshold to generate a *de novo* OTU database. The raw, combined sequences were then mapped to the *de novo* database to generate sequence counts per OTU and sample. OTUs were assigned taxonomic classifications using the RDP classifier (Wang et al., 2007) trained on the Greengenes database (August 2013 version; McDonald et al., 2012). All OTUs classified as mitochondria or chloroplasts were removed from analyses. Samples were then rarefied to 9800 sequences per sample, resulting in a total of 23,782 OTUs in the final data set.

Unless otherwise noted, bacterial sequence data were analyzed using R (Version 3.2.2, R Core Team, 2015). The combined data set was then used to generate three distinct data subsets: All Samples (202 bat skin and environment samples collected from a site where bats were sampled and soil samples that are not paired with bat samples), Bats Only (164 only bat skin samples, no environmental samples or soil samples), and Paired Samples (93 samples where bats and their environments (cave wall) could be sampled together). Within the Bats Only subset, a smaller subset of the 70 *M. lucifugus* samples was generated in order to better describe site and state variation within a single species.

We examined alpha diversity in the Bats Only and Paired Only data sets, respectively, for significant differences across sample type, site, species, and region. We used a custom script in the *mctoolsr* (https://github.com/leffj/mctoolsr), *biom*, and *ggplot*, and *vegan* libraries to calculate Shannon diversity, richness, and Kruskal-Wallis tests of significance. To determine which bacterial OTUs were shared across bat species, the Paired Only samples were used with the core_microbiome.py script in QIIME (http://qiime.org). In order to assess differences in community composition, we used a Bray-Curtis dissimilarity matrix for each of the 4 data subsets (All Samples, Bats Only, and Paired Only, and *M. lucifugus* Only). We used permutational multivariate analysis of variance (PERMANOVA) via the *adonis* function within the *vegan* package to examine the differences between communities using host species, site, and region as explanatory factors. To test for differences in dispersion amongst groups, we used *betadispr* in the *vegan* package. In addition, we used pairwise PERMANOVAs to examine which bat species, sites or regions were driving significant differences. We applied false discovery rate (FDR) corrected *p*-values to the pairwise PERMANOVA tests. The Bray-Curtis dissimilarity matrix was used to create ordinations using non-metric multidimensional scaling (NMDS) for each data set in order to visualize the effects of state, site, and host species on bacterial communities. We used a multiple linear effects model to determine which bacterial classes were driving differences in community composition among bat species, with site as a random effect. Relative abundances of bacterial classes were rank transformed to meet the assumptions of the models. All *p*-values were FDR corrected based on the number of taxa tested (all bacterial classes with mean relative abundances ≥1% in any species).

### Comparison of bats to other vertebrate skin microbiomes

To compare the skin bacteria observed on bats to other vertebrates, we performed a literature search and identified 9 studies representing 4 taxa with the following criteria: (1) targeted 16S rRNA gene for bacteria, (2) comparable high-throughput sequencing techniques within the last 7 years, and (3) a measure of the bacterial abundances on a specific area of skin. We mined the 5–10 most abundant bacterial taxa per host species, and calculated percent relative abundance using available supplemental materials or graphs as provided by published papers. Remaining taxa, excluding the 5–10 most abundant bacterial taxa, were labeled as “Other Classes” or “Other Phyla,” respectively.

## Results

### Sequencing results

The clustering step (>97%) to a *de novo* database ultimately produced 28,487 OTUs from 235 samples. The average number of sequences per sample was 52,250. Samples were rarefied to 9000 sequences per sample, which removed 29 samples from the dataset that had fewer sequences than the threshold and four samples which were negative controls. In total, 202 bat and environment samples achieved high quality sequencing and were included in downstream analyses. (Supplementary Table 1). These samples were used in three data sets: Bats Only (166), Paired Only (93 samples, 19 environmental and 74 bats), *M. lucifugus* only (70) and All Samples (202).

### Bacterial communities inhabiting bat skin

**Figure 1:**
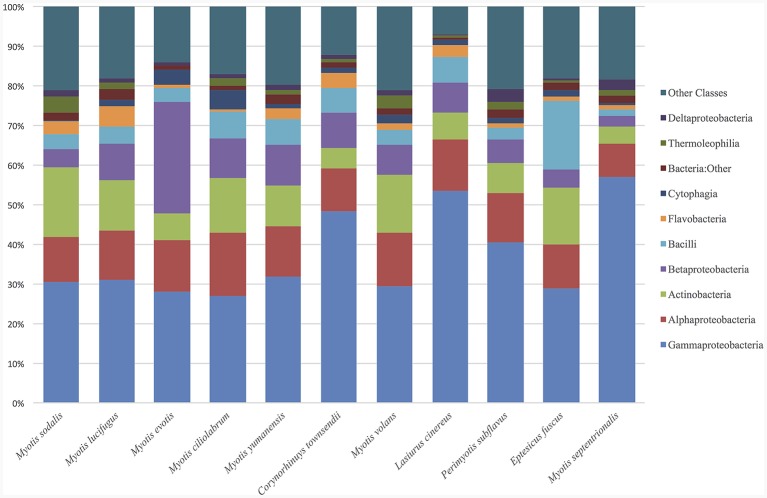
**Relative abundance of bacterial classes in environment and bat skin samples**. The microbial composition by class of bat skin across all species included in the study. Only the top 15 OTUs are represented, which comprise, on average, 89% of all OTUs in the sample.

The top classes represented in the data set for all samples were: Gammaproteobacteria, Alphaproteobacteria, Actinobacteria, Betaproteobacteria, Bacilli, Flavobacteria, Cytophagia, and Thermoleophilia. For both bat and environmental samples, the top 15 OTUs by relative abundance comprise 89% of the sequences. The top OTUs for just bats from all states fall within the classes: Gammaproteobacteria, Alphaproteobacteria, Actinobacteria, Betaproteobacteria, Bacilli, Flavobacteria, Saprospirae, Thermoleophilia, and Clostridia (Figure 1). For bats, there were more than 604 OTU genera represented (with an abundance of more than 0.0001%). Two common genera found on bats are Pseudomonas (9%), and Acinetobacter (5%), and there are many genus-level that remain unclassified at that level such as Xanthamonadaceae: Other (4%), Bacteria: Other (2%), Gammaproteobacteria: Other (2%), Bacilli: Other (2%), Actinomycetales: Other (1.8%), Enterobacteraceae: Other (1.8%), Sphingomonadaceae: Other (1.7%) and Pasteurellaceae: Other (1.5%).

### Comparison to other vertebrates

Across multiple vertebrate taxa, bats share the most classes with other mammals, particularly dogs (Table 2). At the phylum level, Proteobacteria and Bacteroidetes are represented across all taxa, with particularly high abundances (11–61%) of Proteobacteria in all skin microbiomes. Bats and salamanders have skin microbiomes that are largely composed of Proteobacteria, at 65 and 60% relative abundance respectively. At the class level, Gammaproteobacteria are broadly represented across host taxa. Bats and dogs showed a stark difference in their relative abundance of class Gammaproteobacteria, which dominated the bat skin community at more than 40% but was 12.5% in domestic dogs (*Canis familiaris*). Alphaproteobacteria, Betaproteobacteria, and Actinobacteria are also shared across multiple taxa, however in lower abundances overall. One class, Thermoleophilia, was only found on furred mammals (dogs and bats) and Cytophagia was the only class found exclusively on bats. Overall, much of the skin microbial community remains unclassified, even at the class level (between 7 and 59% in the species compared in this data set).

**Table 2:** **Vertebrate skin microbiome**.

**Phylum**	**Class**	**Mammals**				**Fish**	**Amphibians**		**Birds**	
		**Humans^a^**	**Bats^b^**	**Dogs^c^**	**Whales^d^**	**Marine Fishes^e^**	**Frogs^f^**	**Salamanders^g^**	**Turkey Vulture^h^**	**Black Vulture^h^**
Acidobacteria								6		
Actinobacteria		45	13.1	18.5		6	10	1.7	10.6	15.5
	*Actinobacteria*		11.4	6.25		6	5	1.7	10.6	15.5
	*Actinobacteria: Other Classes*			10.25			5			
	*Thermoleophilia*		1.7	2						
Bacteriodetes		5	4.3	7.25	32.6	4	7.2	2	6.6	12.1
	*Bacteriodia*						1		2.3	5.9
	*Bacteriodetes: Other Classes*			7.25		4	3			
	*Cytophagia*		2							
	*Flavobacteriia*		2.3		32.6		1		2.3	6.2
	*Sphingobacteria*						2.2	2		
Chloroflexi										3.9
	*Thermomicrobia*									3.9
Cyanobacteria		9.5				1		2.4	3.7	
Firmicutes		29	8.5	14.75		34		1	39.3	34
	*Bacilli*		6.8	6.5					13.1	9.6
	*Clostridia*		1.7					1	26.2	24.4
	*Firmicutes: Other Classes*			8.25		34				
Fusobacteria				2			1		3	6.1
	*Fusobacteriia*						1		3	6.1
	*Fusobacteria: Other Classes*			2						
Plantomycetes								1.9		
	*Plantomycetacia*							1.9		
Proteobacteria		11	65.3	46.75	36.6	42.5	32.4	60.7	31.2	20.8
	*Alphaproteobacteria*		13.4		1.2	7	0.5	10.7	3.7	2.3
	*Betaproteobacteria*		9.8	21.5	1.5	12.5	27.6		11	5.4
	*Deltaproteobacteria*		1.5			2				
	*Gammaproteobacteria*		40.6	12.25	33.9	21	4.3	50	16.5	13.1
	*Proteobacteria: Other Classes*			13						
Tenericutes				0.5						
	*Mollicutes*			0.5						
Other Phyla		0.5	8.8	10.25	30.8	12.5	49.4	24.3	5.6	7.6
	Other Classes		8.8	12.25	30.8	13.5	49.4	32.7	11.3	7.6

*A summary of the relative percent abundance of bacterial phyla (blue) and classes (yellow) across recently published papers on the skin microbiome of vertebrates. Lighter shades indicates low abundances, darker shades are higher abundances. Bacteriodetes and Proteobacteria are shared across all taxa in their skin microbiome*.

a *Costello et al., 2009*,

b *Avena et al., 2016*,

c *Hoffmann et al., 2014*,

d *Apprill et al., 2014*,

e *Larsen et al., 2013*,

f *Kueneman et al., 2014*,

g *Fitzpatrick and Allison, 2014*,

h *Roggenbuck et al., 2014*.

### Diversity patterns by region, site, and species

In order to examine how diversity varied across species, sites, and regions, Shannon diversity indices were calculated for paired environment and bat samples as well as within bat samples only. For the paired samples across all sites, Shannon diversity of environmental samples was marginally different from the bat skin samples (*p*-value = 0.06), and there are observed differences in alpha diversity metrics between sites and regions. When comparing differences between bats at different sites, there were significant differences in alpha diversity observed among all sites and species in both Shannon diversity and richness (Shannon Diversity: Kruskal-Wallis chi-squared = 20.15, *df* = 10, *p*-value = 0.02, Richness: Kruskal-Wallis chi-squared = 24.70, *df* = 10, *p*-value = 0.005).

### Community composition patterns by region, site, and species

**Figure 2:**
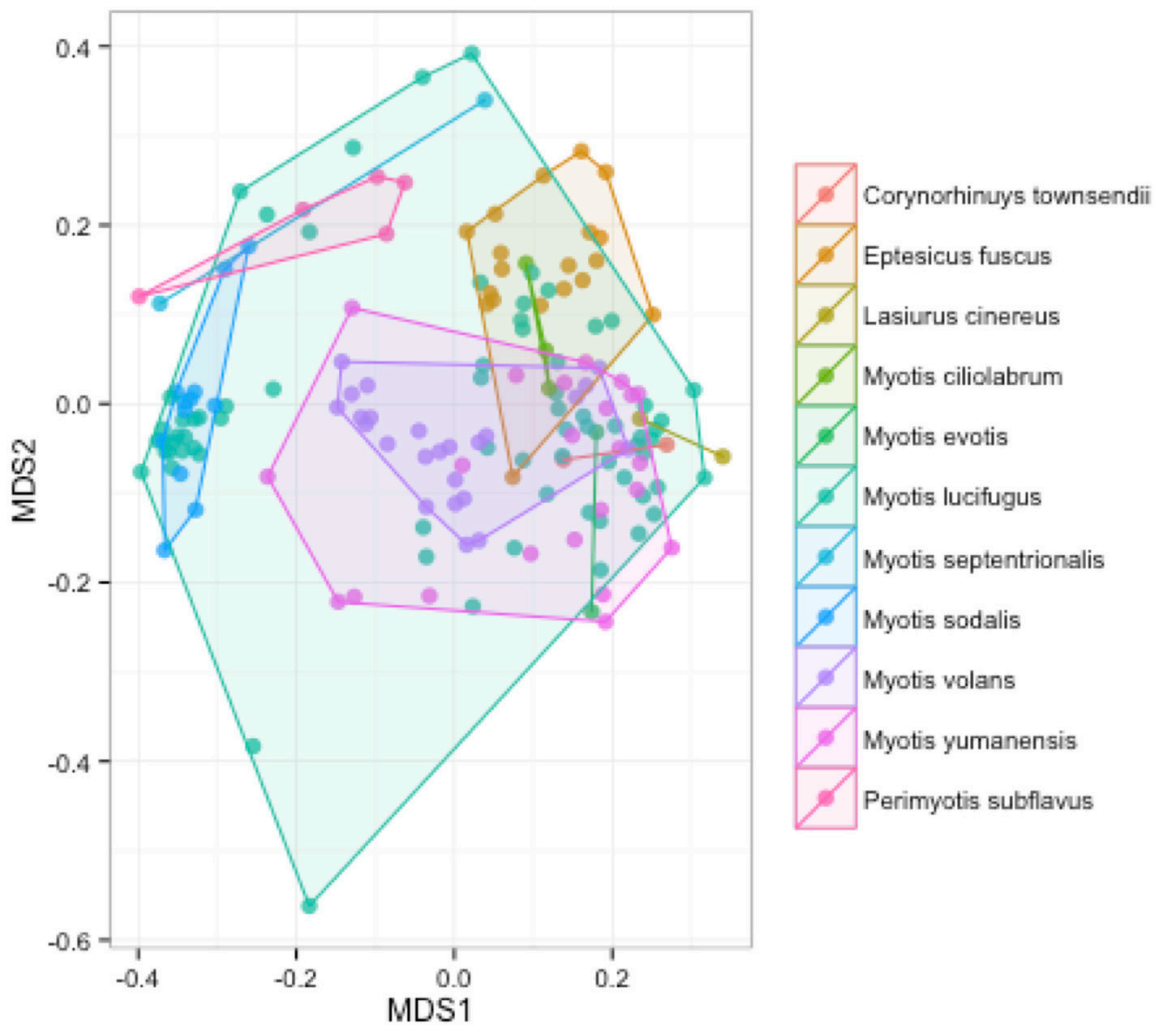
**Differences in community composition of all bat samples by species**. An NMDS ordination of a Bray-Curtis dissimilarity matrix of bacterial communities across all bats in the sample set, colored by species (stress = 0.18). A PERMANOVA test showed differences between species were significant (*R*^2^: 0.15, *p*-value < 0.001).

**Figure 3:**
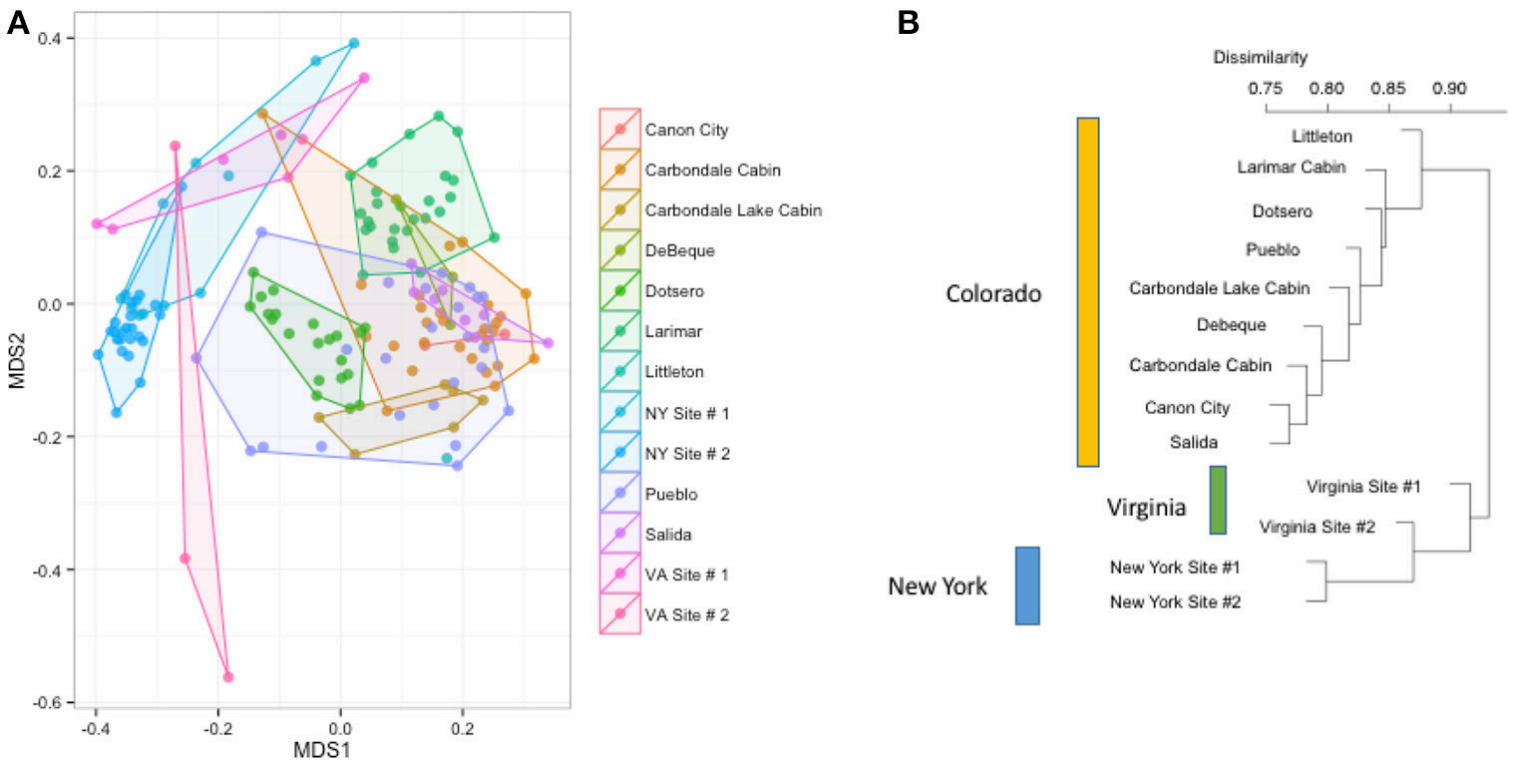
**(A,B)** Differences in bacterial community composition of all bat samples by sample site. NMDS ordination of all bats from Figure 2 colored by site (stress = 0.18). A PERMANOVA analysis indicates that site is a significant driver of community composition (*R*^2^: 0.26, *p*-value < 0.001). At right, the Bray-Curtis dissimilarity between sites plotted as a dendrogram.

**Figure 4:**
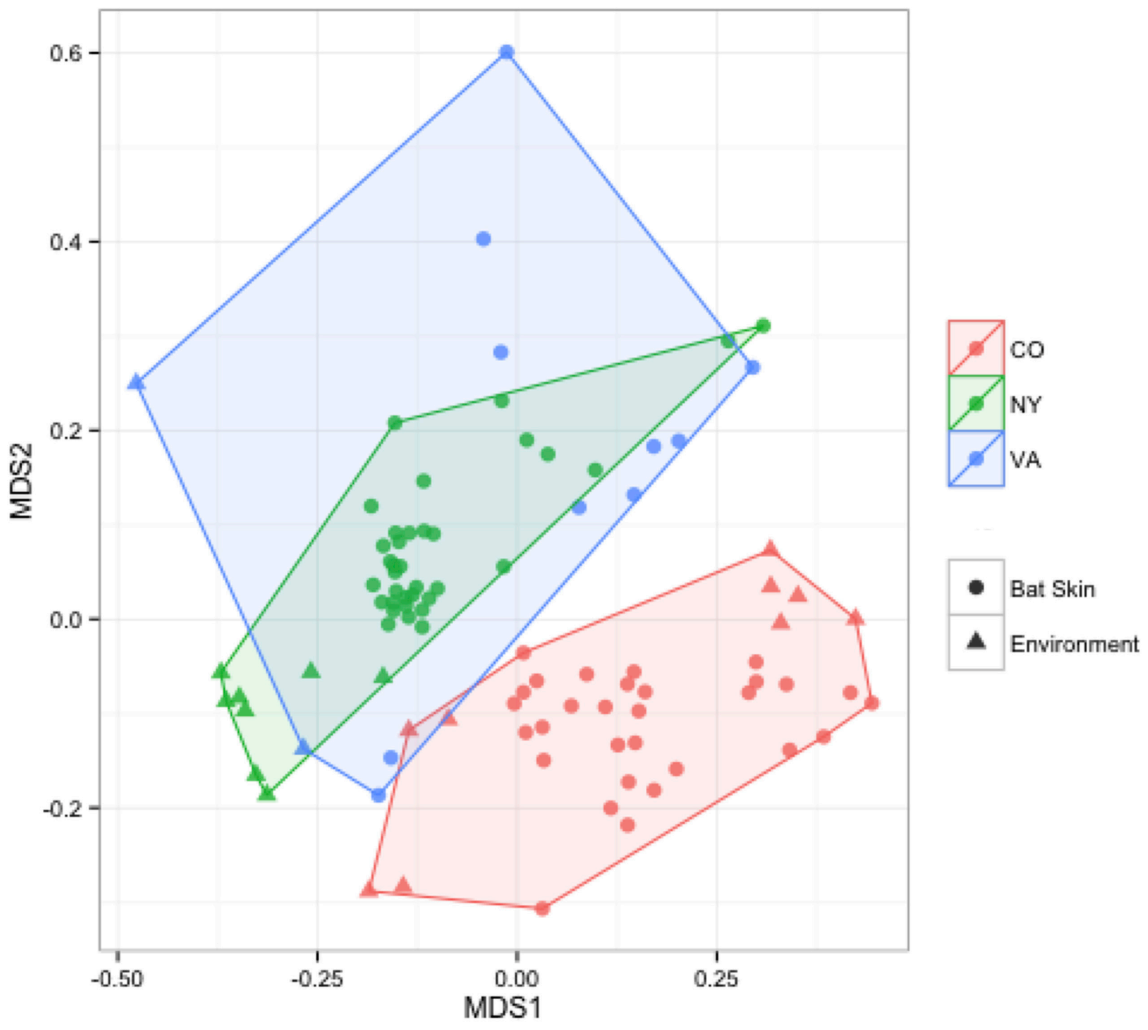
**Comparisons of beta diversity of bat skin by region from paired samples**. An NMDS ordination of paired samples (soil and bat swabs collected from the same location) sampled for this data set, colored by region (stress = 0.18). A PERMANOVA analysis indicates that region is significant in determining the bacterial community composition (*R*^2^: 0.16, *p*-value < 0.001).

We measured patterns in community composition across sample regions, sample sites, and host species (beta diversity) in the Bats Only data set of 164 individuals found across 11 different sites in all 3 regions (NY, VA, and CO). Among the bat samples, seven OTUs were found across 85% of all samples, suggesting that these could be considered as core taxa. They were classified as belonging to the class and family, Gammaproteobacteria: Pasteurellaceae, Gammaproteobacteria: Enterobacteriaceae, Alphaproteobacteria: Sphingomonadaceae, and Betaproteobacteria: Burkholderiales. We used PERMANOVA analyses to assess differences in the bacterial community and find that region, site, and species all significantly explained variability (*p*-value < 0.001 for each variable, *R*^2^ 0.12, 0.27, and 0.15). These differences are apparent when the NMDS plot is colored by species (Figure 2). *Betadispr* analysis of each of the factors (site, sample, and region) within the Bats Only data set showed that there are significantly different dispersion levels for each (each *p*-value < 0.001). To further explore these patterns, we used pairwise PERMANOVAs. Many of the comparisons between community composition were significant across data sets (i.e., Bats Only) and sample groups (i.e., site). (Supplementary Table 2). Using a multiple liner effects model, Betaproteobacteria were found to be the class that had the strongest effect on differences in community composition on bat skin since it was the only one that varied significantly across species (FDR corrected *p*-value = 0.009). The significant (*p*-value < 0.001) differences in beta diversity between sites for the Bats Only data set are shown in an NMDS plot in Figures 3A,B, with an accompanying dendrogram showing the geographic pattern of beta diversity across sites. Lastly, the significant (*p*-value < 0.001) difference in beta-diversity between regions is graphed using an NMDS of the paired samples analysis, with each region highlighted in a different color and each sample type (bat skin vs. environment) represented by a shape (circle or triangle, Figure 4). These results demonstrate a high amount of exchange from the environment (at both the site and regional level) as well as the bat host species in the bacterial communities of bat skin. However, when considering the overall dissimilarities between skin bacterial communities between and within sample site beta diversity and species beta diversity, there is more variation between sites than between species (Figure 6). This suggests that while environmental and host factors are both important in determining the composition of the skin microbiome, differences between sites more strongly predict differences in bacterial communities than differences across host species.

### The influence of region and site bacterial community composition within a single species

**Figure 5:**
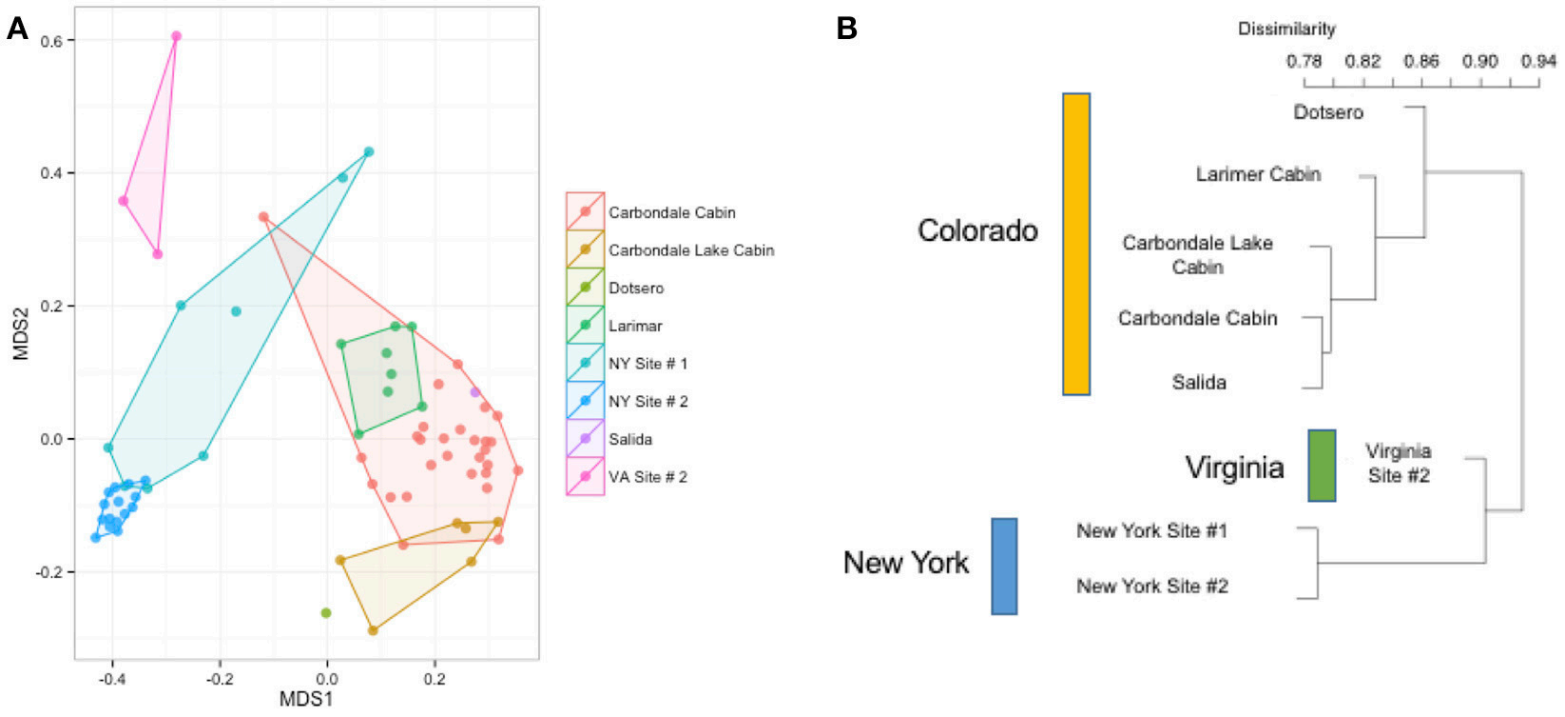
**(A,B)** Differences by site in *Myotis lucifugus.* Within a single species sampled across multiple states (*M. lucifugus*), site is very important in determining the beta-diversity of the bacterial community, as visualized in an NMDS ordination (stress = 0.14). (PERMANOVA *R*^2^: 0.33, *p*-value < 0.001). The clustering of the Virginia samples (upper left, in pink) and the New York samples (center, in blue) also shows the regional signal. At right, a dendrogram of the Bray-Curtis dissimilarity matrix for these sites.

To better understand the differences between the effects of species on the host bat community and the effect of environment (on both the regional and site-specific level), we used a subset of the data set that included a single species. Samples from 70 individuals of *M. lucifugus* from all three states and multiple sites in each state were used for further analysis. Using a Bray-Curtis distance matrix, patterns by site and region were visualized using an NMDS ordination and the dissimilarity patterns between host bacterial communities were analyzed using PERMANOVA (Figure 5A). Site and region were both significant factors for the *M. lucifugus* subset, suggesting that there may be site- and region-specific microbes that influence the development of the microbiome shared within a species (*R*^2^: 0.33, *p*-value < 0.001). The NMDS plot, colored by site and state, shows clear differences in the bacterial communities between different sites where bats were sampled. A dendrogram of the beta diversity dissimilarity shows that the most similar communities are grouped by state (Figures 5A,B).

**Figure 6:**
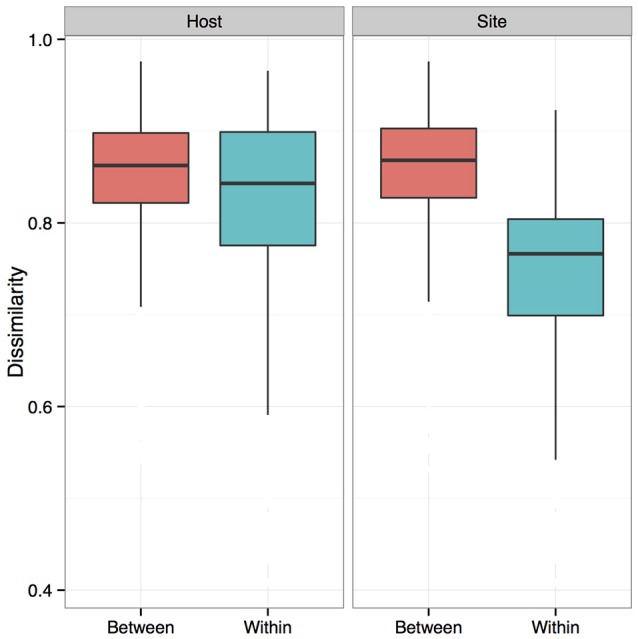
**Dissimilarity analysis of bat skin bacteria between and within host species and sites**. Boxplots showing bacterial community composition dissimilarities between and within samples from the same host species (Host) or sample site (Site). The differences between samples from the same sites and different sites is greater than that between and within species. Boxes represent first quartile medians and third quartile values, and lines represent minimum and maximum values. Dissimilarities were calculated using a Bray-Curtis dissimilarity matrix from square-root transformed OTU relative abundances.

### Differences between the environment and the bat host in paired samples

**Figure 7:**
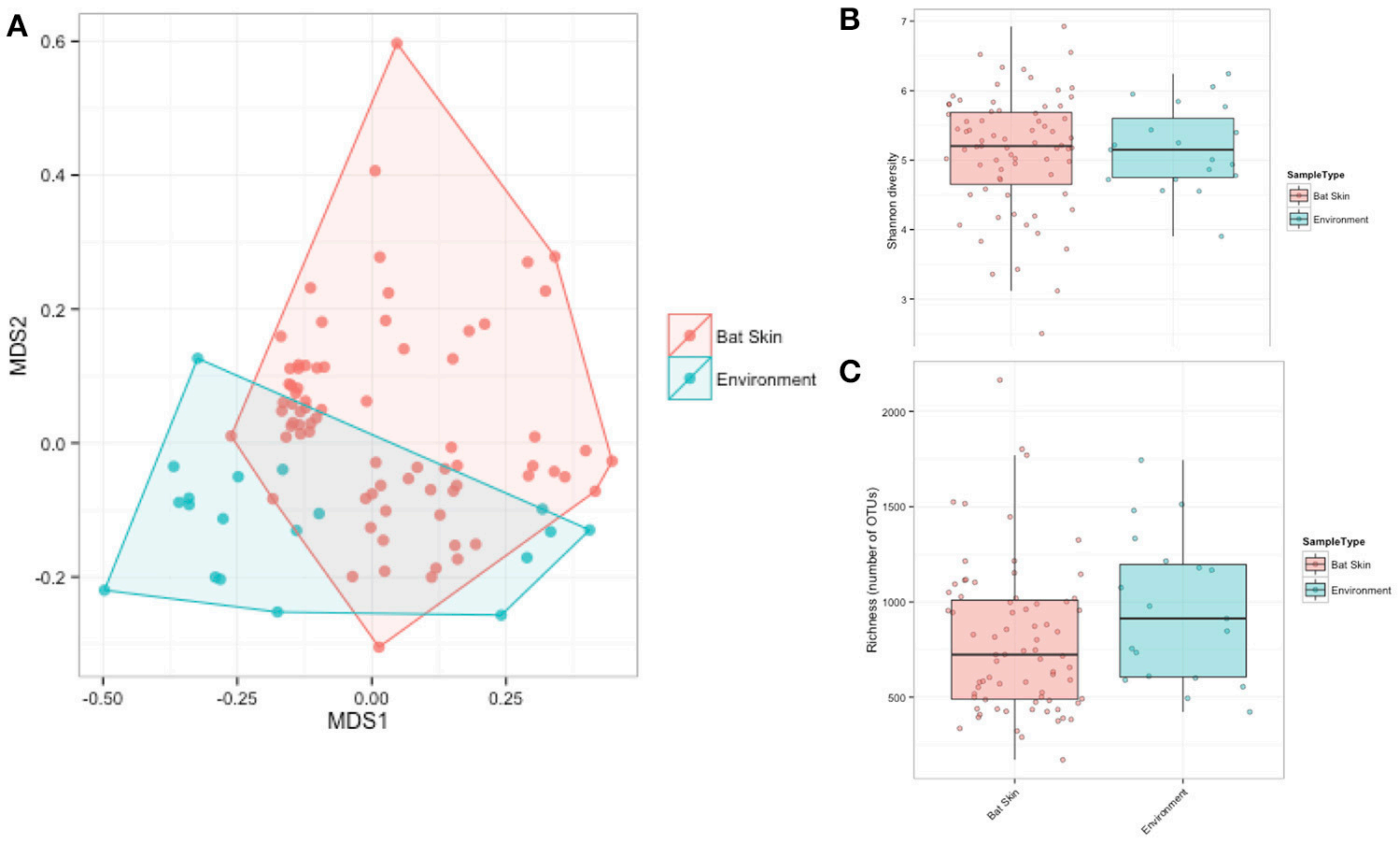
**(A–C)** Comparisons of paired environment and bat samples by alpha and beta diversity metrics. **(A)** Beta diversity of paired environmental samples (blue) and bat skin samples (red) were significantly different (*R*^2^: 0.033, *p*-value < 0.001) as shown in an NMDS ordination (stress = 0.20). **(B)** Shannon diversity and **(C)** richness were not significantly different between the environment and bat skin, with many of the dominant taxa shared between groups. (Kruskal-Wallis test of significance *p* > 0.05).

**Figure 8:**
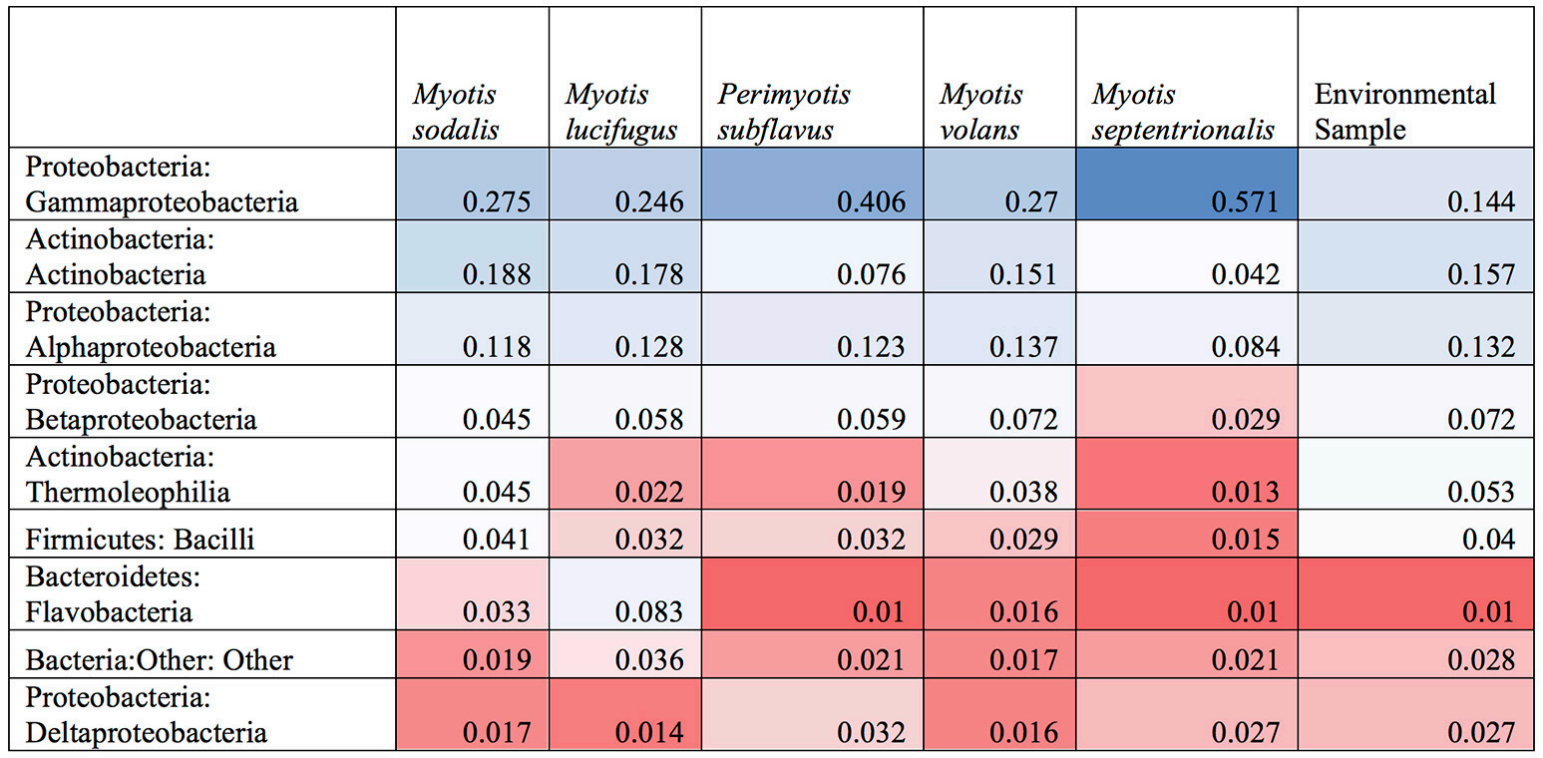
**Heatmap of shared OTUs between bats and environment by class**. A heatmap of the relative abundances if the top ten most common bacterial classes found on paired samples of bat skin and their local environment. Blue indicates high abundance taxa, white indicates moderately abundant taxa, and red indicates less abundant taxa. While the most and least abundant taxa are generally shared between the environment and the bat, bacteria in the classes of Thermoleophilia and Bacilli appear in higher abundances on the bat than in the environment.

A subsample of the data set that included paired environment (cave wall or soil) samples as well as bat samples (skin swabs) for sample site were analyzed to see which taxa were shared amongst the two groups. Soil and bats did not differ significantly in their richness or Shannon diversity (Kruskal-Wallis test of significance) (Figures 7B,C). Based on these results, a heatmap was generated using relative abundances to compare the top 10 classes found on both bats and environment, which shows that Gammaproteobacteria, Alphaproteobacteria, and Actinobacteria are the most abundant and are also shared within both soil and bats (Figure 8). However, a PERMANOVA analysis of the difference in community composition of the microbial communities between bat skin and the environment using a Bray-Curtis dissimilarity matrix found a significant difference (Figure 7A) (*R*^2^ = 0.033, *p*-value < 0.001), suggesting that there are differences below the class level that distinguish soil and bat skin.

### *P. destructans* results

WNS testing yielded five positive individuals of the 68 bats sampled from the eastern US portion of the data set, although only four of these individuals were used in the final analysis. WNS status was not a significant factor in the community composition of bat skin community when comparing either the entire sample set or when subsampling only from within sites with *M. lucifugus* (ANOSIM using QIIME, *p* > 0.05). Supplementary Figure 1 shows PCOA plots of infected and uninfected individuals across all species and within just *M. lucifugus*. There are no strong patterns by infection status.

## Discussion

### Characterizing the bat skin microbiome and their environment

The bat skin microbiome is influenced by both species and the habitat in which the host is found. Many of the same bacterial genera are found in similar abundances across bat species (Figure 1). Many of the bacterial taxa found on bat skin are also dominant taxa in soil and plant material, suggesting that these groups are shared readily between the host and its environment (Figure 8). The community of bacteria found on bat skin contains high diversity, with even the most abundant bacteria comprising less than 10% of the overall sample. *Pseudomonas* (9% of total abundance in the paired bat and environmental data set) is a commonly found genus in the environment, along with *Acinetobacter* (5%) and the plant-associated family *Xanthamonadaceae* (4%). As bats are the most vagile mammal species, it is possible that during activities such as foraging and migration they are acquiring a diverse microbial community from the environment. Two other groups of interest in this data set that are known to be host-associated. *Enterobacteriaceae* (1.8%) is an environmental as well as gut-associated bacterial family and suggests that there may be contamination of the skin with fecal material, which is not surprising given the close association with the roost environment. Previous culture-based studies have also detected *Pseudomonas* on bat skin, and found multiple strains in the *Pseudomonas fluorescens* complex which exhibited anti-fungal properties against *P. destructans* (Hoyt et al., 2015). *Pasteurellaceae* (1.5%) is usually associated with the oral microbiome, suggesting that these microbes may be deposited on the skin during grooming bouts. Both *Enterobacteriaceae* and *Pasteurellaceae* were found to be shared between 85% of all the bat species as part of the core microbiome analysis. Overall, the knowledge of how many of these taxa are specialized to the bat host remains limited by the availability of data for host-associated bacterial genera (Table 2). However, the general pattern emerges that much of the diversity of bacterial taxa is shared between both bats and the environment, and many of those taxa are known to be either common symbionts of other mammalian hosts or found in the environment.

### The bat skin microbiome: special amongst vertebrates?

In a review of the available literature on the skin microbial communities of four mammals (humans, dogs, whales and bats), two birds (turkey vultures and black vultures), two amphibians (wild frogs and red-backed salamanders) and marine fishes, bats share some of the key skin associations with other terrestrial mammals (dogs) but have few bacterial taxa in common across these groups. The important classes shared between dogs and bats include Alphaproteobacteria, Betaproteobacteria, Gammaproteobacteria, Actinobacteria, Bacilli, and Thermoleophilia (Table 2). However, bats and dogs showed a difference in their relative composition of class Gammaproteobacteria, which dominated the bat skin community at more than 40% but was only 12.25% in dogs. Gammaproteobacteria was found in moderately high abundance in whales (33.9%) and 50% in salamanders, and was shared in varying amounts amongst all the wild vertebrates sampled. One class, Thermoleophilia, is only found on bats and dogs, suggesting a relationship to terrestrial mammals, however it is a known soil organism (Suzuki and Whitman, 2012; Crits-Christoph et al., 2013). For all of the vertebrates examined for this study, 7.6%–49.4% of bacteria were not in the most abundant taxa. Although broad comparisons could be made, synthesis across studies was complicated by disparate sampling schemes, analysis types, and OTU reporting methods. For comparative analyses in the future and the ability to understand shared microbial taxa between study groups, it is essential that future studies provide taxa tables to aid in comparative analyses to allow an understanding of broader patterns across taxa.

### Regional, local, and species effects on the host-associated microbial community

Many factors were found to be significant drivers of the host-associated microbial communities of bats. There were significant differences in the community composition of the bacterial communities between the three regions (Colorado, New York, and Virginia) (Figure 4). While there are shared taxa among these three areas, New York and Virginia bats appear to share more in common with each other than Colorado, suggesting a regional pattern of soil- and host- associated microbial communities, though broader regional sampling would be needed to confirm this conclusion. Using the data set containing only bat samples, there are significant differences amongst bacterial OTU abundances (alpha diversity) and between these microbial communities (beta diversity) by both species and site (Figures 1, 3). Patterns of host-associated bacterial communities within a single species (specifically *M. lucifugus)* also show differences between regions and sites. Taken together, this represents a complicated picture of how bats acquire and maintain their skin microbial community. Along with the results of the paired analysis, which suggest that alpha diversity is not different between bats and their environment, this suggests a shared relationship both among bats (inter- and intra-species) and between the bat hosts and their surrounding environment.

### What are the drivers of bat skin microbiome? comparisons to patterns in other taxa

The results from the bat microbial community suggest that many of the bacteria that are dominant in the environment of hibernacula are also shared with bats, which may provide one mechanism for why we see general regionalized patterns as well as site-specific differences as the strongest factors explaining variation in the bat skin microbiome (Figure 8). However, a significant difference in the bacterial community composition amongst the 14 bat species sampled does exist, though less pronounced than the site effects. In amphibians, species tends to be a better predictor of the host-associated skin community rather than site (McKenzie et al., 2012; Kueneman et al., 2014). For a single whale species (*Megaptera novaeangliae*), sampling location is known to be an important factor shaping the host skin community (Apprill et al., 2014). In humans and domestic dogs, we know that the composition of the skin microbial communities can be affected by many factors, such as disease status, temporal variation, and body site location (Costello et al., 2009; Grice et al., 2009; Grice and Segre, 2011; Hoffmann et al., 2014). In homes where domestic dogs and humans share the same environment, many of the bacterial taxa will swap hosts to form a mixed microbial community that is significantly different from other humans or domestic dogs not within the same household (Song et al., 2013). In a recent study of carrion-eating birds, many of the taxa found on the exposed dermis on the heads of two species of vultures closely resembled prey items, as well as taxa that were specific to bird skin (Roggenbuck et al., 2014). The results from the bat microbial community suggest that many of the bacteria that are dominant in the environment of hibernacula are also shared with bats, which may provide one mechanism for why we see general regionalized patterns as well as site-specific differences as the strongest factors explaining variation in the bat skin microbiome (Figure 8). However, host species effects are also present, suggesting that the bat skin microbiome is influenced by complex and interacting factors. Broader studies that examine whether there are generalizable patterns that drive vertebrate skin microbiome communities are needed.

### Presence of *P. destructans* on bat skin and the future of the bat skin microbiome

For this study, all of the bats sampled were captured at the end of the summer, during the fall swarming period, or at the very start of hibernation. The pathogen only begins to invade and colonize the dermis of the host during hibernation (Langwig et al., 2015) when the temperature of the skin is lowered for sustained periods. None of the bats included in this study were visibly infected, and *P. destructans* loads were low in positive individuals (Supplementary Figure 1). Our dataset does not, therefore, permit a robust investigation of the relationship between *P. destructans* and the bat skin bacterial community.

Bats share much of their abundant microbial taxa with the environment, and this may drive the patterns observed in the alpha- and beta-diversity differences between sampling sites. There is more variation observed between site beta-diversity than between species (Figure 6). To a lesser extent, but still significant, species play an important role in determining the composition of the bat skin microbiome. Overall, the complex factors regulating the bat microbiome suggest that the environment and host factors are important, but more research is necessary to understand the relative contributions and functions of these host-associated microbial communities. We are just beginning to understand the patterns of microbial diversity on bat hosts, and understanding the ecology of these associations will build a foundation for future work on the influences on host health and interactions with the environment.

Overall, our understanding of host-associated skin microbial communities is still in its infancy. Compared to the amount of research available on the human microbiome, the microbiome of animals, and particularly skin, remains relatively underexplored, and many of the patterns that we observe need to be rigorously evaluated. The skin microbial community is not as abundant nor as diverse as the gut community, and therefore requires particular care when collecting and processing samples from the field. The emergence of new fungal diseases, several of which invade the skin of their host and cause morbidity and mortality, highlights the importance of fungi for understanding microbiomes (Fisher et al., 2012). Understanding the patterns by which these communities assemble can shed light on how to approach these infectious diseases from a microbiome perspective and possibly enhance treatment of these diseases.

## Data accessibility

All sequence data (16S rRNA bacterial marker gene reads) will be submitted to the European Bioinformatics Institute (EBI) and be openly accessible upon publication. Complete metadata will accompany all sequence information.

## Author contributions

VM and CA designed the study. Sample collection was conducted by CA, KL, KP, WF, and AK. Sample processing was conducted by CA, HA, and JF. LP, JL, and CA analyzed the data and generated figures. Writing was done by CA and VM. All authors contributed toward the editing of the manuscript.

## Funding

This collaborative work was supported by funds from multiple sources including NSF grants (DEB: 1146284 to VM and DEB: 1115895 to WF, JF, and AK), a grant from the John S. Templeton Foundation to VM, a grant from Bat Conservation International to CA and VM, and a University of Colorado EBIO departmental summer research grant to CA. Publication of this article was funded by the University of Colorado Boulder Libraries Open Access Fund.

### Conflict of interest statement

The authors declare that the research was conducted in the absence of any commercial or financial relationships that could be construed as a potential conflict of interest.
