# Supplementary material for: Deconstructing the Bat Skin Microbiome: Influences of the Host and the Environment
Source: Front Microbiol. 2016 Nov 17;7:1753. doi: 10.3389/fmicb.2016.01753 (PMC5112243; doi:10.3389/fmicb.2016.01753)
Supplement: Supplementary file 3 [file Image1.PDF]

Supplementary Material

Deconstructing the Bat Skin Microbiome: Influences of the Host and the Environment

*Christine V. Avena, Laura Wegener Parfrey, Jonathan W. Leff, Holly M. Archer, Winifred F. Frick, Kate E. Langwig, A. Marm Kilpatrick, Karen E. Powers, Jeffrey T. Foster, and Valerie McKenzie

***Correspondence**:
Christine Avena
Christine.avena@gmail.com

1 Supplementary Data

Supplemental Table 1: OTU Table of all samples in study. *See attached table (Excel file).*

2 Supplementary Figures and Tables

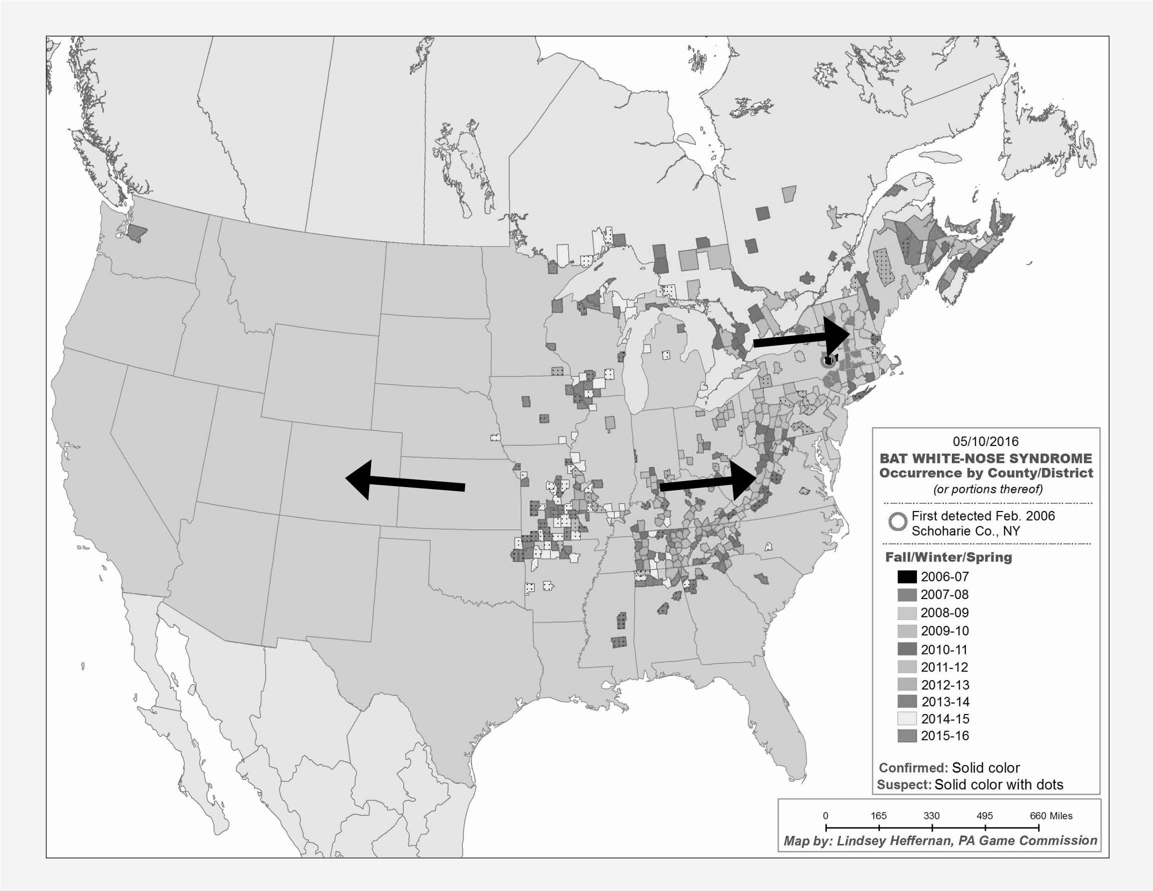

Supplementary Figure 1: Current WNS spread map for the United States and Canada, provided by the USGS/USFWS and made by Lindsey Heffernan of the PA Game Commission. Study areas are marked with a black arrow. Colorado does not contain any positive *Pd* samples as of winter 2015.
